# Retraction Note: Progranulin causes adipose insulin resistance via increased autophagy resulting from activated oxidative stress and endoplasmic reticulum stress

**DOI:** 10.1186/s12944-022-01746-3

**Published:** 2022-12-13

**Authors:** Qinyue Guo, Lin Xu, Huixia Li, Hongzhi Sun, Jiali Liu, Shufang Wu, Bo Zhou

**Affiliations:** 1 Department of Respiratory and Critical Care Medicine, the First Affiliated Hospital of Xi’an Jiaotong University, 277 Yanta West Road, Xi’an, 710061 Shaanxi China; 2 Department of Endocrinology, the Affiliated Guangren Hospital of Xi’an Jiaotong University, Xi’an, 710004 Shaanxi China; 3 Key Laboratory of Environment and Genes Related to Diseases, Medical School of Xi’an Jiaotong University, Xi’an, 710061 Shaanxi China; 4 Clinical Laboratory, the Second Affiliated Hospital of Xi’an Jiaotong University, Xi’an, 710061 Shaanxi China; 5 Center for Translational Medicine, the First Affiliated Hospital of Xi’an Jiaotong University, Xi’an, 710061 Shaanxi China

**Retraction Note: Lipids Health Dis 16, 25 (2017)**

**https://doi.org/10.1186/s12944-017-0425-6**

The Editor-in-Chief has retracted this article. Following publication, concerns were raised regarding the reliability and availability of the data reported in this article. The authors were not able to share the original data. An investigation by the First Affiliated Hospital Xi’an Jiaotong University confirmed that the original data were not available and recommended the retraction of the article.

Authors Huixia Li and Jiali Liu agree with this retraction. The remaining authors have not responded to correspondence regarding this retraction.

